# Unrecognized Cystocele

**Published:** 1830-04-01

**Authors:** 


### CASE OF CYSTOCELE WHICH WAS NOT RECOGNIZED FOR A GREAT NUMBER OF YEARS.*

Several cases of hernia of the bladder are on record, one of the most remarkable in the works of Mr. Pott, but no doubt many have occurred which were either not understood at all, or mistaken for other varieties of hernia. Mr. Gilbert Burnett related an interesting case of cystocele in the early part of the present season at the Westminster Medical Society, and another instance of this affection is recorded by M. Ribell of Perpignan, in the Archives Générales. It is useful on occasions to be aware of the symptoms and character of rare diseases, as no practitioner can be sure that a case of the kind will not happen to himself, and his character will rise or fall in proportion to his acquaintance with or ignorance of the subject.

---

* Archives Générales, Nov. 1829.

*Case.* M. P***, of Prats de Mollo, fifty-eight years of age, had suffered for upwards of thirty years from difficulty of making water, without much pain, but a necessity of micturition every two or three hours. Prior to the first appearance of this complaint, he had suffered from an eruption on the leg which was healed by proper treatment, and after the cure of which the urinary symptoms commenced. M. P*** had likewise suffered since birth from a large inguinal hernia on the left side, and since the age of fifteen or eighteen from a smaller one on the right. Neither were treated with a truss until the patient's marriage at the age of 25, when one was applied which made but ineffectual compression. The difficulty of making water began about the age of 28, but for five years the patient took no medical advice on the subject. Being then rather alarmed at its continuance he repaired to Perpignan, and was sounded by several surgeons. They informed him that the bladder was healthy, but that the urethra presented numerous strictures which occasioned great difficulty to the passage of the instrument, and had been occasioned by the disappearance of the cutaneous affection on the leg. We need not stop to notice the treatment founded on the foregoing diagnosis, suffice it to say that it did no good, and the patient abandoned the regulars to put himself into the hands of a Quack. He promised largely, and gave him strong doses of the corrosive sublimate with the effect of "horribly fatiguing" the stomach. We should here observe that the patient had never suffered from syphilis or gonorrhœa.

On the 22d of August, 1828, having been troubled for five days with more than ordinary dysuria, M. P*** was suddenly seized in the evening with complete retention of urine. After much straining he voided a little pure blood, and subsequently some blood mixed with a little urine and much mucus. Our author was summoned from Perpignan, but owing to the distance was unable to arrive until after the lapse of 24 hours. He had then just made water, though with difficulty, and was free from any fever or excitement. On a subsequent visit our author determined to satisfy himself of the state of the bladder and urethra, but on introducing an instrument, he was not a little surprised to find that it could be passed without difficulty or obstruction of any description, and half a wine-glass full of limpid urine evacuated. It was quite clear from this fact that there was not, and never had been, a stricture of the urethra. But what was there then, to account for the dysuria of thirty years' duration? By dint of close inquiry and accurate examination the following particulars were ascertained.

1mo, The difficulty of making water was always greatest when the inguinal hernia on the left side was most voluminous.

2ndo, If the nisus of micturition occurred at the moment when the hernia was small, the urine escaped in a free stream, but if during its evacuation the hernia escaped or enlarged, the flow of the urine was abruptly checked.

3tio, Experience had taught the patient, and without his knowing how or why, that in order to make water easily, he should raise the hernia and make the neck the most dependent part.

4to, After making the patient retain his water as long as possible (scarcely four hours) and keeping him on his legs in order that the hernia might protrude to the utmost, the catheter was introduced and about four spoonsfull of water only abstracted; this too could not be effected till the hernia had been reduced.

5to, A forcible injection of two wine-glasses-ful of water into the bladder produced the following results:—it excited a very strong desire to make water, the patient not being accustomed to retain so much liquid in the bladder at a time: it produced a very sensible enlargement of the inguinal hernia though the patient continued recumbent, and it occasioned a distinct sense of fluctuation in the hernial tumour towards its neck.

6to. The hernia on the right side (an intestino-omental one) was readily and completely reducible.

7mo. The left, on the contrary, was irreducible. It appeared to contain intestine towards its lower part, and the testicle seemed to be united to the gut. The neck of the tumour, when grasped between the finger and thumb, gave the sensation of comprising within it two lubricated membranes slipping on each other.

From the foregoing premises, M. Ribell concludes that his patient is affected with a congenital hernia on the left side, originally intestinal; that this hernia not being restrained by any pressure has insensibly enlarged, and involved the bladder; and finally that the latter viscus has contracted adhesions in its new situation, and is consequently irreducible. We perfectly agree with the well-informed narrator of the case, which is a very satisfactory one. It seems rather singular, that although a fluctuation was felt at the neck of the hernial tumour when the bladder became distended, no mention is made of *pressure* on this part evacuating the organ. In the few cases of which we have read, this symptom was particularly noticed, and in one, if we remember right, the patient told his surgeon that the "urine came from his rupture, for he could not piss unless he pressed upon his groin." With regard to the methodus medendi, we fear that the mischief has proceeded too far in the present case to admit of relief from art. The adhesions of the bladder and the diminution of its calibre, the necessary consequences of its long continuance in the sac, are accidents or alterations for which we have no remedy. Pressure is obviously now out of the question.

